# Advanced age and female sex protect cerebral arteries from mitochondrial depolarization and apoptosis during acute oxidative stress

**DOI:** 10.1111/acel.14110

**Published:** 2024-02-21

**Authors:** Charles E. Norton, Rebecca L. Shaw, Safa, Beyoncé Dockery, Timothy L. Domeier, Steven S. Segal

**Affiliations:** ^1^ Department of Medical Pharmacology and Physiology University of Missouri Columbia Missouri USA; ^2^ Dalton Cardiovascular Research Center Columbia Missouri USA; ^3^ Department of Biomedical Sciences University of Missouri Columbia Missouri USA; ^4^ Department of Biomedical, Biological and Chemical Engineering University of Missouri Columbia Missouri USA; ^5^ Department of Nutrition and Exercise Physiology University of Missouri Columbia Missouri USA

**Keywords:** aging, apoptosis, calcium, cell death, mitochondria, oxidative stress, reactive oxygen species

## Abstract

Aging increases reactive oxygen species (ROS) which can impair vascular function and contribute to brain injury. However, aging can also promote resilience to acute oxidative stress. Therefore, we tested the hypothesis that advanced age protects smooth muscle cells (SMCs) and endothelial cells (ECs) of posterior cerebral arteries (PCAs; diameter, ∼80 μm) during exposure to H_2_O_2_. PCAs from young (4–6 months) and old (20–26 months) male and female C57BL/6 mice were isolated and pressurized (~70 mm Hg) to evaluate cell death, mitochondrial membrane potential (ΔΨ_m_), ROS production, and [Ca^2+^]_i_ in response to H_2_O_2_ (200 μM, 50 min). SMC death and ΔΨ_m_ depolarization were greater in PCAs from males vs. females. Aging increased ROS in PCAs from both sexes but increased SMC resilience to death only in males. Inhibiting TRPV4 channels with HC‐067047 (1 μM) or Src kinases with SU6656 (10 μM) reduced Ca^2+^ entry and SMC death to H_2_O_2_ most effectively in PCAs from young males. Activating TRPV4 channels with GSK1016790A (50 nM) evoked greater Ca^2+^ influx in SMCs and ECs of PCAs from young vs. old mice but did not induce cell death. However, when combined with H_2_O_2_, TRPV4 activation exacerbated EC death. Activating Src kinases with spermidine (100 μM) increased Ca^2+^ influx in PCAs from males vs. females with minimal cell death. We conclude that in males, chronic oxidative stress during aging increases the resilience of cerebral arteries, which contrasts with inherent protection in females. Findings implicate TRP channels and Src kinases as targets to limit vascular damage to acute oxidative injury.

## Graphical abstract

Schematic depicts mechanisms of protection from acute oxidative stress in posterior cerebral arteries. Advanced age and female sex limit Src‐dependent Ca^2+^ influx through transient receptor potential vanilloid (TRPV4) channels, attenuate depolarization of mitochondrial membrane potential (ΔΨ_m_), and reduce smooth muscle cell death resulting from exposure to H_2_O_2_. Image created with Biorender.

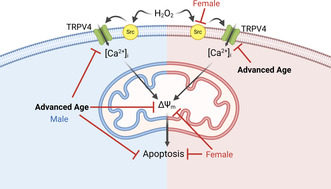

**Abbreviations**
ACh: acetylcholine
[Ca^2+^]_i_
: intracellular calcium
EC: endothelial cell
NE: norepinephrine
PCA: posterior cerebral artery
ROS: reactive oxygen species
SMC: smooth muscle cell
TBI: traumatic brain injury
TRPV4: transient receptor potential vanilloid 4
ΔΨ_m_
: mitochondrial membrane potential

## 1 INTRODUCTION

The brain requires a continuous blood supply, which the cerebral vasculature redistributes according to local neuronal activity and metabolic demand. During aging, compromised cerebral blood flow as a consequence of vascular pathology is linked to the onset and progression of age‐related cognitive impairment and dementia (Cortes‐Canteli & Iadecola, 2020; Ungvari et al., 2010). Vascular apoptosis has long been associated with aging (Cliff, 1970) and leads to vessel rarefaction (De Silva & Faraci, 2020). The risk for ischemic stroke is also elevated in elderly populations (Coco et al., 2016). In addition, adults ≥75 years old have the highest risk for morbidity and mortality from traumatic brain injury (TBI) (Thompson et al., 2006). In each case, aging, stroke, and TBI augment the production of reactive oxygen species (ROS) which damage affected neuronal and vascular cells (Abdul‐Muneer et al., 2015; Li et al., 2003; Rodrigo et al., 2013; Ungvari et al., 2010).

Acute exposure to H_2_O_2_ leads to apoptosis of cerebral vascular smooth muscle (SMCs) and endothelial cells (ECs) by inducing intracellular Ca^2+^ ([Ca^2+^]_i_) overload (Norton et al., 2022). In nonvascular cells, [Ca^2+^]_i_ overload can lead to depolarization of mitochondrial membrane potential (ΔΨ_m_) and initiate apoptosis (Kroemer et al., 2007; Singh et al., 2007), but how SMCs and ECs in cerebral arteries regulate ΔΨ_m_ in response to acute oxidative stress is unknown. Vascular ROS levels increase with aging (Izzo et al., 2021), and females possess mechanisms that can protect them from oxidative stress and cell death (Dantas et al., 2002; Vina et al., 2005). Nevertheless, the effects of advanced age and sex on apoptosis and ΔΨ_m_ depolarization in cerebral arteries during ROS exposure remain to be defined.

In mouse superior epigastric arteries (SEAs), advanced age increases SMC resilience to H_2_O_2_ by reducing Ca^2+^ entry through transient receptor potential vanilloid 4 (TRPV4) channels, with SEAs of females inherently more resilient to H_2_O_2_ than those from males (Norton et al., 2019). Whether such age and sex differences in resilience are manifest in the cerebral vasculature during aging is unknown, as is the mechanism of vascular TRPV4 channel activation by ROS. Oxidative stress activates TRPV4 channels through Src family kinases (Wegierski et al., 2009), which can mediate apoptosis in epithelial cells (Chan et al., 2009). Whether Src family kinases contribute to cerebral vascular cell death induced by H_2_O_2_ exposure is undefined.

To provide insight into how age and sex affect cell death and ΔΨ_m_ depolarization in cerebral arteries during ROS exposure, we tested the hypotheses that advanced age and female sex: (1) increase resilience of mouse posterior cerebral arteries (PCAs) to acute oxidative stress imposed by H_2_O_2_ and (2) reduce TRPV4‐dependent Ca^2+^ entry mediated by Src kinases. We evaluated the effects of advanced age and sex on the susceptibility of SMCs and ECs in PCAs to cell death during 50 min exposure to 200 μM H_2_O_2_. These criteria for H_2_O_2_ exposure were based on values associated with ischemia/reperfusion injury in the rat forebrain (Hyslop et al., 1995) and defined in prior studies of mouse PCAs (Norton et al., 2022). The present findings reveal that advanced age and female sex reduce ΔΨ_m_ depolarization and cell death during H_2_O_2_ exposure by limiting Ca^2+^ entry through TRPV4 channels in response to Src kinase activation.

## 2 RESULTS

### 2.1 Advanced age and female sex increase resilience of PCAs to acute oxidative stress

**FIGURE 1:**
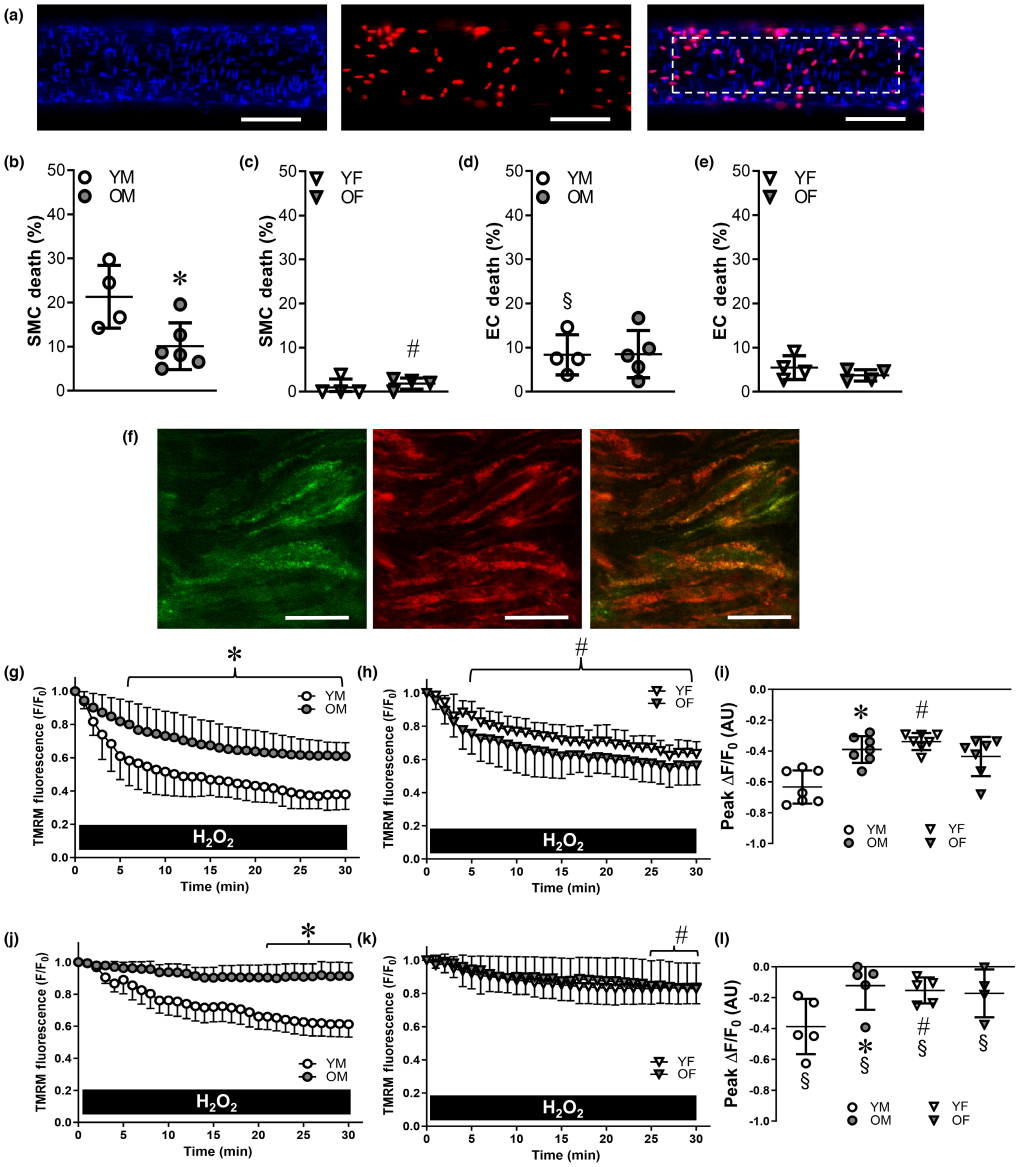
Advanced age and female sex protect against H_2_O_2_‐induced cell death and ΔΨ_m_ depolarization in posterior cerebral arteries (PCAs). (a) Hoechst 33342 stains all nuclei (left, blue), propidium iodide stains nuclei of dead cells (center, red) in a PCA from an old male mouse following H_2_O_2_ exposure; merged image (right). Dotted rectangle indicates ROI and contains ∼50 SMCs (thin vertical nuclei) and 50 ECs (oval horizontal nuclei); scale bars = 50 μm. Small circular nuclei are from adventitial cells injured during dissection. (b) Percentage of dead SMCs in PCAs from young males (YM) and old males (OM). (c) Percentage of dead SMCs in PCAs from young females (YF) and old females (OF). (d) Percentage of dead ECs in PCAs from YM and OM. (e) Percentage of dead ECs in PCAs from YF and OF. (f) Confocal imaging of SMC layer for TMRM (left, green), mitotracker (center, red), and merged image (orange‐yellow, right) in an isolated pressurized PCA from a young male; scale bars = 10 μm. (g) Changes in ΔΨ_m_ (TMRM fluorescence; F/F_0_) during H_2_O_2_ exposure in PCAs from YM and OM. (h) Changes in ΔΨ_m_ during H_2_O_2_ exposure in PCAs from YF and OF. (i) Peak change in ΔΨ_m_ in response to H_2_O_2_ in PCAs from each group. (j) Changes in ΔΨ_m_ during H_2_O_2_ exposure in endothelial tubes from YM and OM. (k) Changes in ΔΨ_m_ during H_2_O_2_ exposure in endothelial tubes from YF and OF. (l) Peak change in ΔΨ_m_ in response to H_2_O_2_ in endothelial tubes from each group. Data are from individual experiments with means ± SD; *n* = 4–7 vessels/group. In (k), data for OF V overlie YF V. *p < 0.05, OM vs. YM. ^#^ *p* < 0.05, YF vs. YM. ^§^ *p* < 0.05 EC vs. intact PCAs. Statistics: two‐way anova with Bonferroni post hoc tests.

Staining SMC and EC nuclei identified live and dead cells in pressurized PCAs (Figure 1a). Following H_2_O_2_ exposure (200 μM, 50 min), SMC death was lower for old vs. young male mice (Figure 1b). In young females, SMC death was significantly lower vs. young males, however, aging did not alter SMC death in females (Figure 1c). EC death was significantly lower than SMC death in PCAs from young male mice and neither age nor sex affected EC death (Figure 1d,e). In the absence of H_2_O_2_, SMCs and ECs in PCAs have <1% death under these experimental conditions (Norton et al., 2022).

Depolarization of ΔΨ_m_ is a critical step in apoptosis in response to H_2_O_2_ (Singh et al., 2007). In all PCAs, H_2_O_2_ depolarized ΔΨ_m_ and this fall in TMRM fluorescence (corresponding to a decrease in F/F_0_) was significantly attenuated in vessels from old vs. young males (Figure 1g,i). Depolarization of ΔΨ_m_ was also significantly less in PCAs from young females vs. young males (Figure 1h,i) with no further effect of age in females, nor were there differences between old males and old females. To focus on ΔΨ_m_ in ECs, TMRM experiments were performed in endothelial tubes (Figure S1). Depolarization of ΔΨ_m_ during H_2_O_2_ exposure was attenuated in EC tubes from old vs. young males (Figure 1j) and was minimal in EC tubes from females of either age (Figure 1k). Regardless of age or sex, ΔΨ_m_ depolarization was less in EC tubes (Figure 1l) compared to intact PCAs (Figure 1i). Initial fluorescence values (*F*_0_) were not different between groups. In the absence of H_2_O_2_, ΔΨ_m_ remained stable in both preparations for the protocol duration (Figure S2). The protonophore FCCP was used as a positive control in PCAs from young males and elicited pronounced depolarization of ΔΨ_m_ over 30 min (Δ*F*/*F*_0_ = 0.682).

### 2.2 Effects of age and sex on cerebral and cerebrovascular ROS production

**FIGURE 2:**
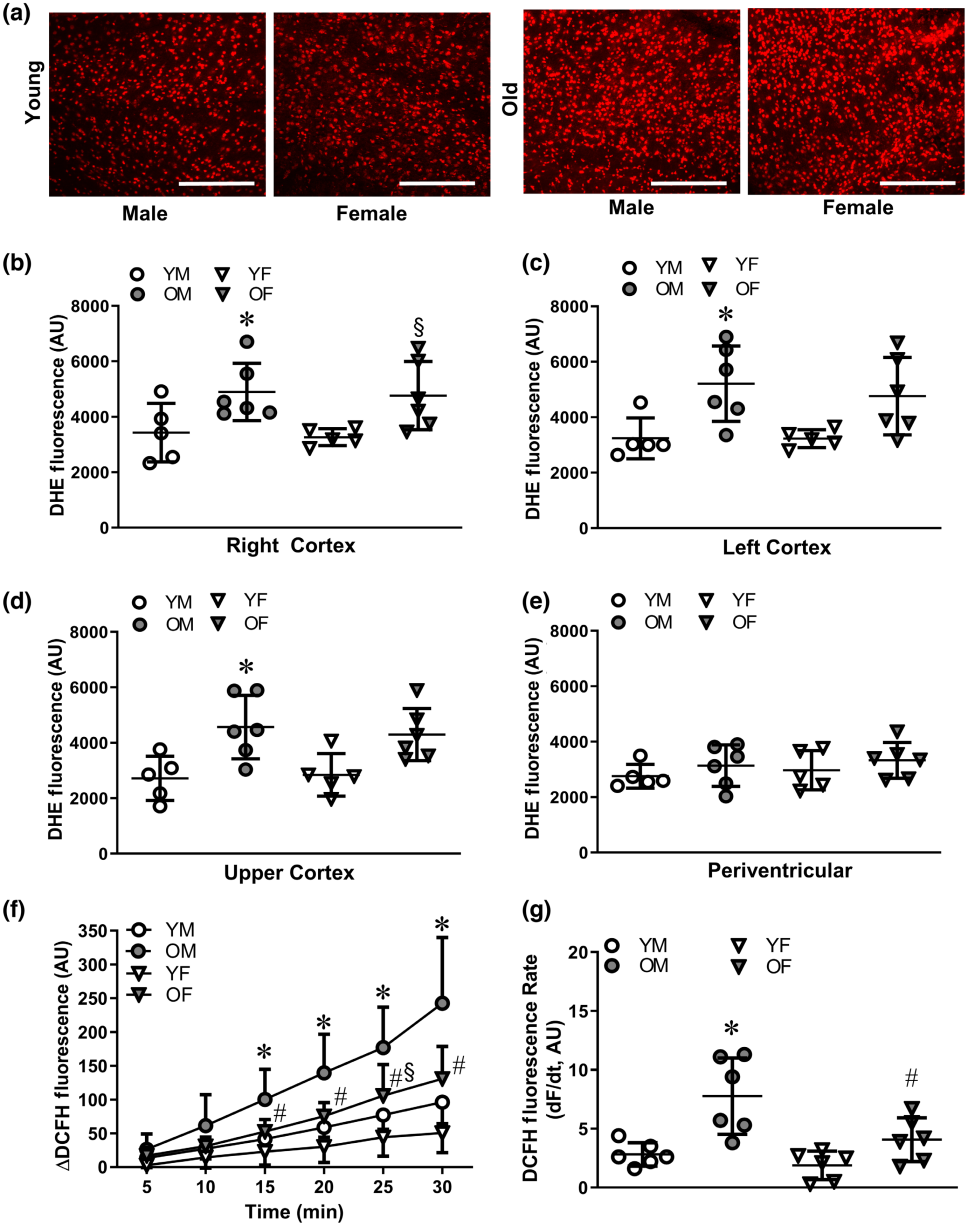
Advanced age increases ROS production in brain tissue. (a) Representative dihydroethidium (DHE) fluorescence for quantification of ROS throughout coronal brain slices of the lower left cortex from (young males) YM, old males (OM), young females (YF), and old females (OF); scale bars = 200 μm. DHE fluorescence (Arbitrary units, AU) in the (b) lower right cortex, (c) lower left cortex, (d) upper cortex, and (e) periventricular region of white matter. (f) Summary data for ROS production over time (ΔDCFH fluorescence) in PCAs from YM, OM, YF, and OF. (g) Rate of DCFH fluorescence accumulation (dF/d*t*) in vessels from each group. Data are from individual experiments with means ± SD; *n* = 5–6 samples/group. *p < 0.05, OM vs. YM. ^§^ *p* < 0.05 OF vs. YF. ^#^ *p* < 0.05 OF vs. OM. Statistics: two‐way anova with Bonferroni post hoc tests.

Throughout the cerebral cortex, aging significantly increased DHE fluorescence (i.e., superoxide production) as an index of ROS generation in brain tissue sections from males, with a similar trend in females (Figure 2a–d). In the periventricular region, ROS levels were not different between sex or age groups (Figure 2e). When averaged across all four brain regions, aging significantly increased ROS production in males [AU: young = 3036 ± 635 (*n* = 5), old = 4455 ± 864 (*n* = 6); *p* = 0.024] and females [young = 3117 ± 391 (*n* = 5), old = 4300 ± 786 (*n* = 6); *p* = 0.014], with no difference between sexes in either age group.

To evaluate vessel wall H_2_O_2_ production, DCFH fluorescence accumulation over time was quantified in isolated pressurized PCAs. For young mice, no differences were detected in H_2_O_2_ generation between the sexes. However, advanced age significantly augmented H_2_O_2_ production in PCAs from both sexes, with a greater effect in males (Figure 2f,g).

### 2.3 Advanced age reduces H_2_O_2_‐induced Ca^2+^ entry through TRPV4 channels

**FIGURE 3:**
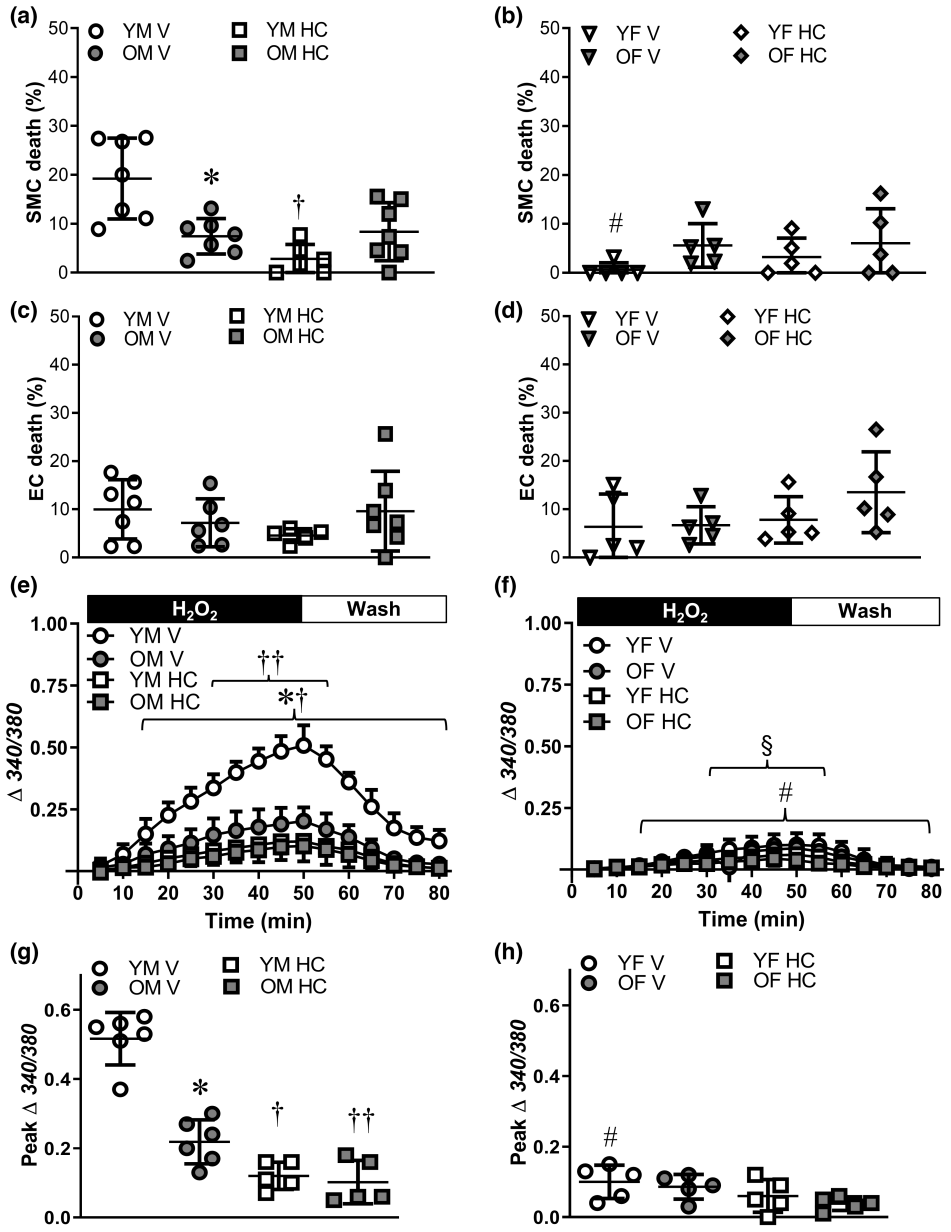
Inhibition of TRPV4 channels reduces SMC death in posterior cerebral arteries (PCAs) of young males. SMC death to H_2_O_2_ in the presence of the TRPV4 channel inhibitor HC‐067047 (HC, 1 μM) or its vehicle (V, 0.1% EtOH) in PCAs from (a) young males (YM) and old males (OM) and (b) young females (YF) and old females (OF). EC death in the presence of HC or vehicle in PCAs from (c) YM and OM and (d) YF and OF. [Ca^2+^]_i_ responses (Fura 2) in PCAs from (e) YM and OM and (f) YF and OF during 50 min H_2_O_2_ and 30 min wash in the absence (V) and presence of HC. Peak changes in [Ca^2+^]_i_ in the absence and presence of HC in PCAs from (g) YM and OM and (h) YF and OF. Data are from individual experiments with means ± SD; *n* = 5–7 vessels/group. In (f), data for OF HC overlie YF V, YF HC, and OF V. *p < 0.05, OM vs. YM in V. ^#^ *p* < 0.05, YF vs. YM in V. ^§^ *p* < 0.05 OF vs. OM in V. ^†^ *p* < 0.05, HC vs. V in PCAs from YM. ^††^ *p* < 0.05, HC vs. V in PCAs from OM. Statistics: two‐way anova with Bonferroni post hoc tests.

During exposure of PCAs to H_2_O_2_, Ca^2+^ influx through TRPV4 channels contributes to SMC apoptosis (Norton et al., 2022). For PCAs from young males, TRPV4 channel inhibition with HC‐067047 (1 μM) significantly reduced SMC death (Figure 3a). In contrast, HC‐067047 had no effect on the prevailing low SMC death in PCAs from old males. Inhibition of TRPV4 channels also did not significantly alter the low prevailing SMC death in young or old females (Figure 3b). There were no differences in EC death between age groups, sexes, or HC‐067047 treatment (Figure 3c,d).

For PCAs from young males, H_2_O_2_ exposure led to a progressive rise in [Ca^2+^]_i_ over 50 min that nearly recovered during the 30 min wash in control PSS; advanced age reduced the maximal [Ca^2+^]_i_ response by ~60% (Figure 3e). TRPV4 channel inhibition with HC‐067047 attenuated the rise in [Ca^2+^]_i_ during H_2_O_2_ in PCAs from males with a greater effect in young vs. old (Figure 3e,g); differences between age groups were eliminated in the presence of HC‐067047. In PCAs from female mice, the [Ca^2+^]_i_ response to H_2_O_2_ was inherently lower in both young and old vs. male counterparts (Figure 3f,h) and TRPV4 channel inhibition was without effect. In the absence of H_2_O_2_, [Ca^2+^]_i_ in PCAs remains stable throughout the 80 min protocol (Norton et al., 2022).

### 2.4 Activation of TRPV4 channels promotes EC death during H_2_O_2_ exposure

**FIGURE 4:**
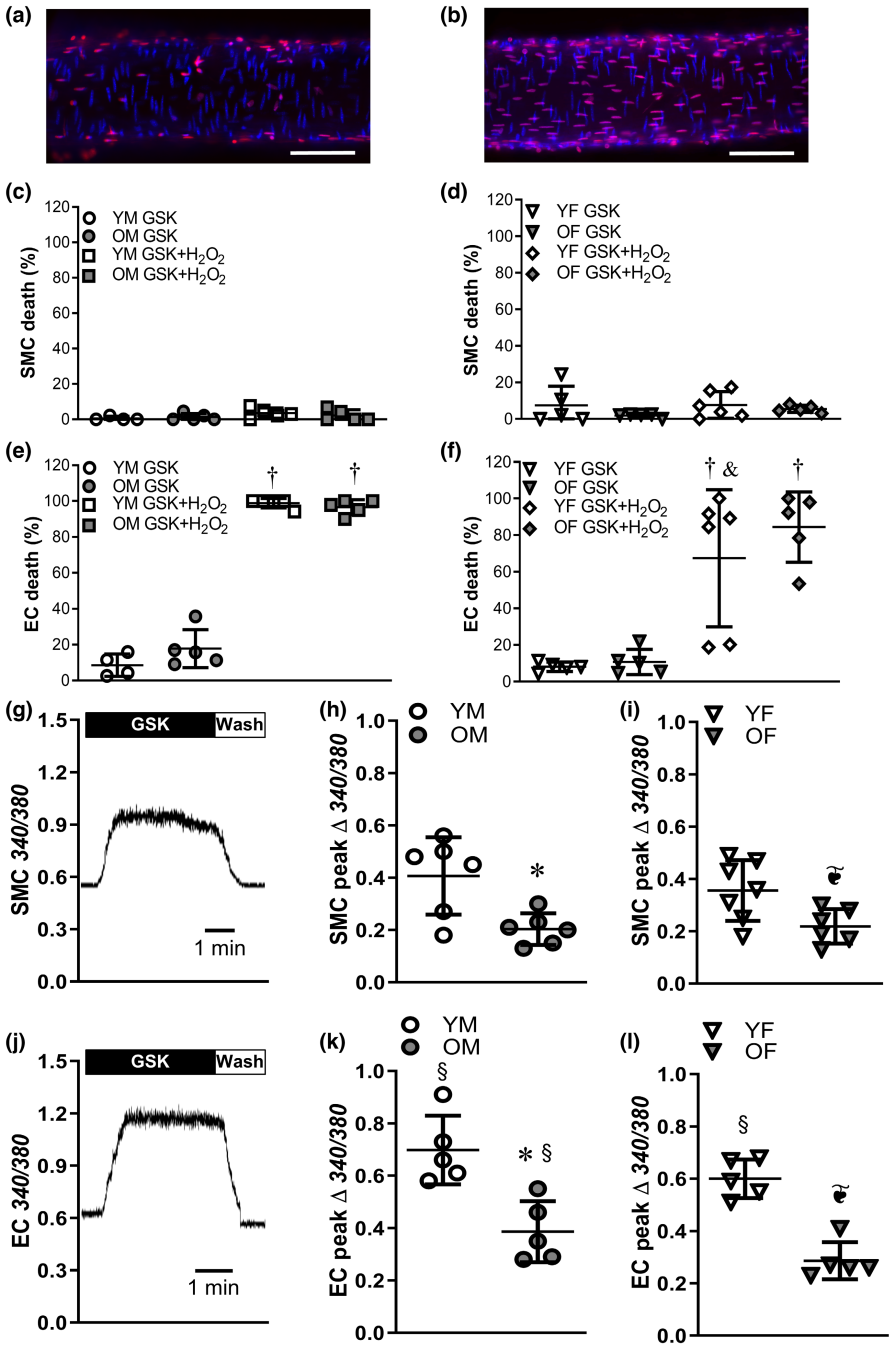
Stimulation of TRPV4 channels during H_2_O_2_ exposure augments EC death. Representative nuclei staining in posterior cerebral arteries (PCAs) from YM mice treated for 50 min with (a) GSK 1016790A (GSK, 50 nM) and (b) with GSK + H_2_O_2_; note dramatic increase in red EC nuclei scale bars = 50 μm. (c) SMC death in PCAs from young males (YM) and old males (OM) following GSK or GSK + H_2_O_2_. (d) SMC death for young females (YF) and old females (OF). (e) EC death in PCAs from YM and OM in the presence of GSK or GSK + H_2_O_2_. (f) EC death for YF and OF. (g) Representative Fura 2 trace in a PCA from YM mouse (primarily reflects SMC [Ca^2+^]_i_) during exposure to the TRPV4 agonist GSK 1016790A (GSK, 50 nM) and wash. (h) Peak change in SMC [Ca^2+^]_i_ in response to GSK in PCAs from YM and OM. (i) Peak change in SMC [Ca^2+^]_i_ in response to GSK in PCAs from YF and OF. (j) Representative Fura 2 trace in an endothelial tube from YM during exposure to GSK and wash. (k) Peak change in [Ca^2+^]_i_ to GSK in ECs of endothelial tubes from YM and OM. (l) Peak change in EC [Ca^2+^]_i_ to GSK for YF and OF. Data are from individual experiments with means ± SD; *n* = 5–6 vessels/group. ^†^P < 0.05, GSK + H_2_O_2_ vs. GSK alone. ^&^ *p* < 0.05, YF vs. YM in GSK + H_2_O_2_. *p < 0.05, OM vs. YM. ^❦^ *p* < 0.05, OF vs. YF. ^§^p < 0.05 EC vs. intact PCAs. Statistics: two‐way anova with Bonferroni post hoc tests.

To determine if direct activation of TRPV4 channels independent of H_2_O_2_ could induce cell death, PCAs were exposed to the TRPV4 agonist GSK 1016780A (50 nM) for 50 min. TRPV4 stimulation did not induce SMC death in PCAs from either males (Figure 4c) or females (Figure 4d) and killed 10%–20% of ECs in males (Figure 4e) and females (Figure 4f) of either age group. Consistently, exposure to GSK alone evoked minimal change in ΔΨ_m_ in PCAs from young males (Figure S3; Δ*F*/*F*_0_ = 0.08 ± 0.09). We then tested whether GSK in combination with H_2_O_2_ would augment cell death. Surprisingly, GSK + H_2_O_2_ resulted in minimal SMC death in males which was less than H_2_O_2_ alone (compare Figure 4c to Figure 1b). In contrast, the combination of GSK + H_2_O_2_ killed nearly all ECs in PCAs from males (Figure 4e) and most ECs in PCAs from females (Figure 4f). In PCAs from young males, neither removal of extracellular Ca^2+^ nor inhibition of caspases with Z‐VAD affected minimal SMC death to H_2_O_2_ + GSK while removal of extracellular Ca^2+^ attenuated EC death to GSK + H_2_O_2_ by ~80% and caspase inhibition reduced EC death by ~50% (Figure S4).

To evaluate how aging alters Ca^2+^ entry through TRPV4 channels, Ca^2+^ responses to GSK 1016780A were measured in intact PCAs [primarily reflecting SMC [Ca^2+^]_i_ (Norton & Segal, 2018)]. The peak [Ca^2+^]_i_ response was reached in ∼90 s (Figure 4g) and the time to maximal response did not differ between groups [young males = 84 ± 31 s, old males = 95 ± 25 s, young females = 88 ± 15 s, old females = 96 ± 23 s (*n* = 6–7/group)]. GSK‐induced Ca^2+^ entry was greater in PCAs from young vs. old males (Figure 4h). Aging also attenuated GSK‐induced Ca^2+^ entry in PCAs from females (Figure 4i). There were no differences between sexes in [Ca^2+^]_i_ responses to GSK.

Because quantification of [Ca^2+^]_i_ in the intact vessel preferentially measures SMC [Ca^2+^]_i_, additional experiments were performed to evaluate the effect of GSK in EC tubes. Although EC tube [Ca^2+^]_i_ responses did not differ between sexes, aging attenuated the maximal increase in [Ca^2+^]_i_ in both males (Figure 4k) and females (Figure 4l). Furthermore, the magnitude of [Ca^2+^]_i_ responses to GSK was consistently greater in EC tubes vs. intact PCAs for all groups.

In EC tubes from PCAs of males and females, there was a trend for H_2_O_2_ exposure to increase [Ca^2+^]_i_ to a greater extent in young vs. old but these differences were not statistically significant and [Ca^2+^]_i_ recovered during washout (Figure S5). Inclusion of GSK during H_2_O_2_ exposure led to a greater increase in the initial [Ca^2+^]_i_ response of all groups which continued to rise during H_2_O_2_ exposure and resulted in greater peak [Ca^2+^]_i_ responses vs. GSK alone. For all groups, [Ca^2+^]_i_ declined during washout but remained elevated at the end of the protocol.

### 2.5 Src kinases contribute to greater SMC death in PCAs from young males

**FIGURE 5:**
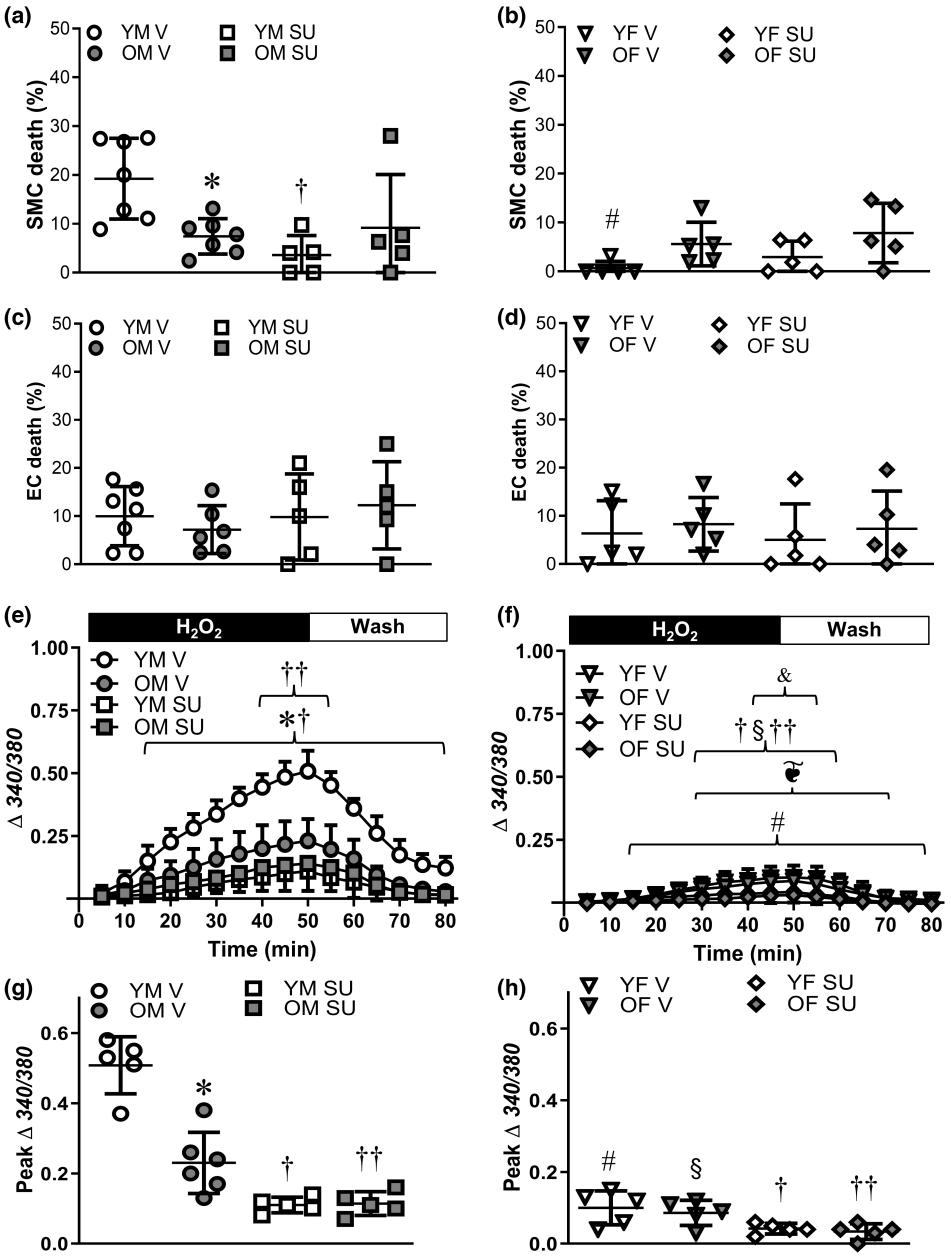
Src family kinases contribute to greater cell death in PCAs of young males. SMC death following 50 min H_2_O_2_ exposure in the presence of the Src kinase inhibitor SU6656 (SU, 10 μM) or its vehicle (V, 0.1% EtOH in physiological salt solution [PSS]) in PCAs from (a) young males (YM) and old males (OM) and (b) young females (YF) and old females (OF). EC death in the presence of SU or vehicle in PCAs from (c) YM and OM and (d) YF and OF. [Ca^2+^]_i_ responses during 50 min exposure to H_2_O_2_ in PCAs from (e) YM and OM and (f) YF and OF during 30 min wash in the absence (V) and presence of SU. Peak changes in PCA [Ca^2+^]_i_ in the absence and presence of SU in (g) YM and OM and (h) YF and OF. Data are from individual experiments with means ± SD; *n* = 5–7 vessels/group. In (f), data for OF V overlie YF V and OF SU overlie OF V. *p < 0.05, OM vs. YM in V. ^†^ *p* < 0.05 SU vs. V in young. ^††^ *p* < 0.05, SU vs. V in old. ^#^ *p* < 0.05, YF vs. YM in V. ^§^ *p* < 0.05, OF vs. OM in V.^❦^ *p* < 0.05, YF vs. YM in SU. ^&^ *p* < 0.05, OF vs. OM in SU. ^§^ *p* < 0.05 EC vs. intact PCAs. Statistics: two‐way anova with Bonferroni post hoc tests.

Src family kinases can be activated by oxidative stress and activate TRPV4 (Wegierski et al., 2009). Inhibition of Src kinases with SU6656 (10 μM) significantly reduced SMC death from H_2_O_2_ exposure in PCAs from young, but not old males (Figure 5a). In PCAs from female mice, Src kinase inhibition had no effect on the low prevailing SMC death (Figure 5b). There was no effect of SU6656 on the low prevailing EC death in vessels from any group (Figure 5c,d).

Src kinase inhibition with SU6656 attenuated the increase in [Ca^2+^]_i_ during H_2_O_2_ exposure in PCAs from males with far greater effect in PCAs from young vs. old (Figure 5e,g). In PCAs from females, the inherently lower [Ca^2+^]_i_ response to H_2_O_2_ was further reduced by Src kinase inhibition (Figure 5f,h) with significant differences persisting between sexes.

**FIGURE 6:**
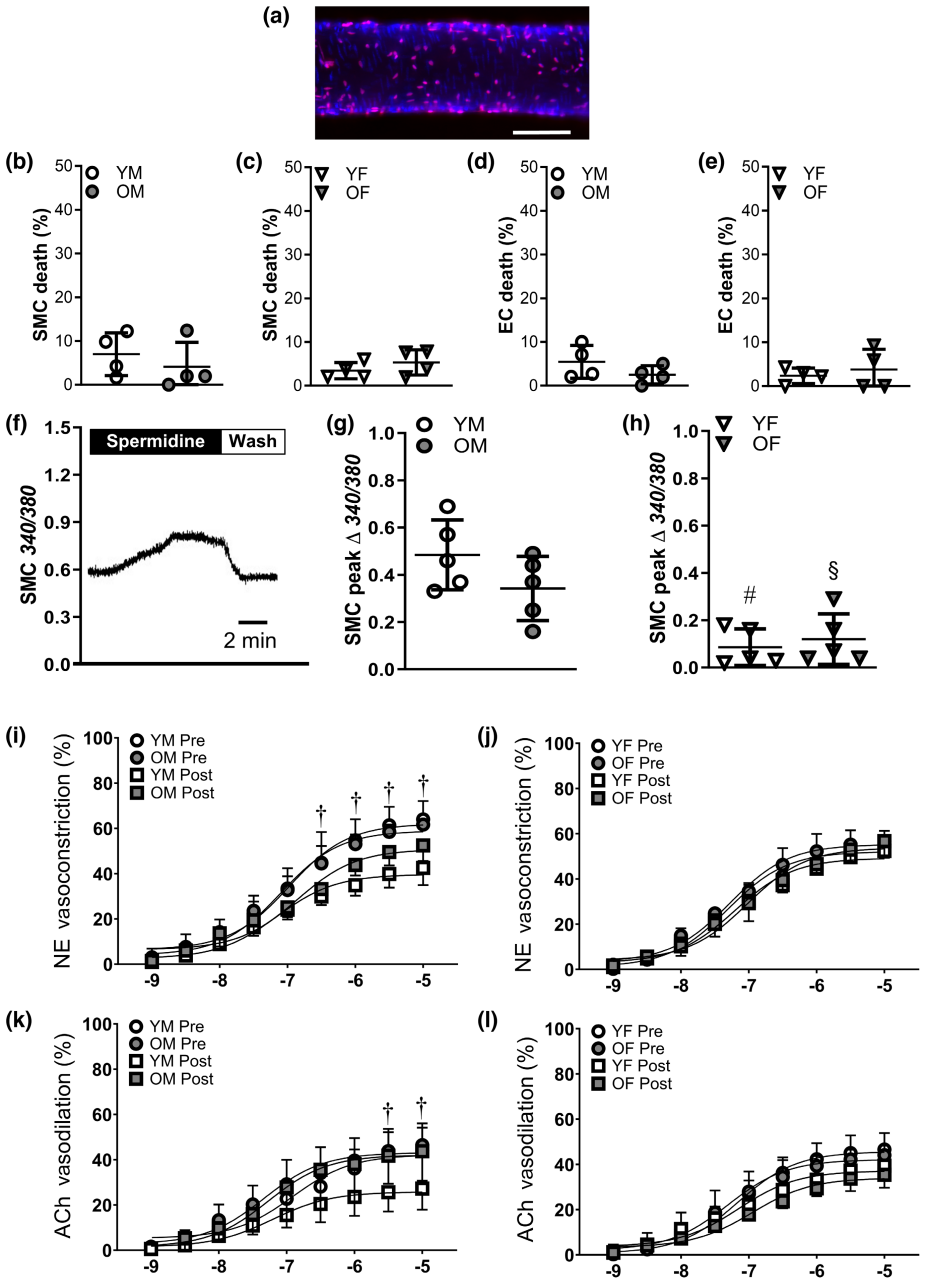
Src kinase activation does not evoke cell death. (a) Representative nuclei staining in a posterior cerebral artery (PCA) from an old female (OF) mouse treated with spermidine (100 μM) for 50 min; scale bar = 50 μm. (b) Smooth muscle cells (SMC) death in PCAs from young males (YM) and old males (OM). (c) SMC death in PCAs from yound females (YF) and old females (OF). (d) Endothelial cell (EC) death in PCAs from YM and OM. (e) EC death from YF and OF. (f) Representative Fura 2 trace in a PCA from YM mouse during exposure to the Src kinase agonist spermidine (100 μM) and wash. (g) Peak change in SMC [Ca^2+^]_i_ in response to spermidine in PCAs from YM and OM. (h). Peak change in SMC [Ca^2+^]_i_ in response to spermidine in PCAs from YF and OF. Concentration–response curves for vasoconstriction to norpinephrine (NE; log M) in PCAs from (i) YM and OM and (j) YF and OF before (pre) and after (post) H_2_O_2_ exposure. Concentration–response curves for vasodilation to acetylcholine (ACh; log M) in PCAs from (k) YM and OM and (l) YF and OF before and after H_2_O_2_ exposure. Data are from individual experiments with means ± SD; *n* = 4–5 vessels/group. In (i), data for OM preoverlie YM Pre; in (j), data for OF SU overlie YF Pre, YF Post, and OF Pre; in (k), data for OM postoverlie YM Pre and OM Pre; in (l), data for OF preoverlie YF Pre. ^#^ *p* < 0.05, YF vs. YM. ^&^ *p* < 0.05, OF vs. OM. ^†^ *p* < 0.05, pre vs. post in YM. Statistics: two‐way anova with Bonferroni post hoc tests.

To further characterize the role of Src kinases in mediating cell death, we activated Src kinases with spermidine (Rossini et al., 2023). Spermidine (100 μM; 50 min) evoked minimal (<10%) SMC death in young and old males (Figure 6b) and females (Figure 6c). EC death was also low for males and females from both age groups (Figure 6d,e). In agreement with the low cell death evoked by spermidine, we saw a minimal change of ΔΨ_m_ in PCAs from young males in response to activation of Src kinases (Figure S6; Δ*F*/*F*_0_ = 0.08 ± 0.11).

Spermidine increased [Ca^2+^]_i_ and reached a maximal response in ~6 min (Figure 6f). Although there was a trend (*p* = 0.15) for Ca^2+^ entry to be reduced in old vs. young males, it was not statistically significant (Figure 6g). The [Ca^2+^]_i_ responses to spermidine were significantly reduced in both young and old females vs. male counterparts (Figure 6h). Treatment with SU6656 reduced the [Ca^2+^]_i_ response to spermidine in young males by ~80% confirming that the effects of spermidine are mediated by Src kinases (Figure S7). Furthermore, TRPV4 inhibition diminished the [Ca^2+^]_i_ response to spermidine by ~50%.

### 2.6 Advanced age and female sex maintain vasomotor control following H_2_O_2_ exposure

Prior to experimentation, the spontaneous resting tone was ~25%, and vasoconstriction to norepinephrine (NE) was similar between groups. Following H_2_O_2_ exposure, vasoconstriction to NE was impaired in young but not old males (Figure 6i) and maintained in females (Figure 6j). Similarly, vasodilation to acetylcholine (ACh) was selectively impaired following H_2_O_2_ exposure in young males (Figure 6k,l). In PCAs from young males, Src kinase inhibition resulted in significantly greater responses to NE and ACh following H_2_O_2_ exposure (Figure S8). Inhibition of TRPV4 channels eliminated differences in vasomotor responses to NE and ACh following H_2_O_2_ exposure, however, it significantly reduced the initial vasodilation to ACh (Figure S8).

## 3 DISCUSSION

This study evaluated the resilience of SMCs and ECs in cerebral arteries from male and female mice to acute oxidative stress induced by exposure to H_2_O_2_. In contrast to the general consensus that aging increases susceptibility to apoptosis in multiple cell types (Behrens et al., 2011; Cooper, 2012), the present findings demonstrate that advanced age increases resilience of cerebral artery vascular SMCs in males to acute oxidative stress challenge and that females have inherent resilience to SMC death. Our data illustrate that, in PCAs from old mice or female mice, depolarization of ΔΨ_m_ and Ca^2+^ entry through TRPV4 channels induced by H_2_O_2_ is diminished compared to young male counterparts. Our findings further support the role of Src family kinases in transducing the signal between oxidative stress and TRPV4 activation. Resolving that inhibition of TRPV4 channels or Src kinases attenuates SMC death to H_2_O_2_ exposure identifies mechanisms of protection that can limit damage to the cerebral vasculature during acute oxidative stress.

### 3.1 Female sex and advanced age protect cerebral arteries from acute oxidative stress

Previously, it was unknown whether advanced age or female sex alters susceptibility of SMCs and ECs to ROS within the cerebral vasculature. The present data from PCAs supplying the brain are consistent with findings from SEAs supplying skeletal muscle (Norton et al., 2019) in that (1) ECs are more resilient to injury from H_2_O_2_ than SMCs in vessels from male mice; (2) SMCs in vessels from female mice are intrinsically protected from H_2_O_2_; and (3) advanced age enhances resilience of SMCs of male mice to H_2_O_2_ (Figure 1). However, even though EC death was also attenuated by advanced age in SEAs (Norton et al., 2019), EC death in PCAs did not differ between old and young mice due to their inherently low death. The differences between these arterial beds suggest that cerebral arterial ECs may have inherently greater resilience to oxidative stress compared to ECs in skeletal muscle arteries.

Intrinsic apoptosis was identified as the mechanism of cell death evoked by H_2_O_2_ in PCAs based on findings that caspase inhibition prevented cell death during H_2_O_2_ exposure, as did removal of extracellular Ca^2+^ to prevent intracellular (and mitochondrial) Ca^2+^ overload (Norton et al., 2022). Depolarization of ΔΨ_m_ secondary to Ca^2+^ overload is a key step in apoptosis (Kroemer et al., 2007; Singh et al., 2007), but the effect of H_2_O_2_ on ΔΨ_m_ in cerebral arteries was unknown. Our findings in PCAs of young and old males and females illustrate that H_2_O_2_ elicited a progressive loss of in ΔΨ_m_ through 30 min and that ΔΨ_m_ depolarization was greater in males vs. females (Figure 1). Advanced age limited ΔΨ_m_ depolarization in PCAs from males but not the inherently protected females. In agreement with the lower EC death observed in response to H_2_O_2_, we observed significantly less depolarization of ΔΨ_m_ in endothelial tubes compared to SMCs of intact arteries across both age groups and sexes.

### 3.2 Advanced age leads to chronic oxidative stress in the brain

Although ROS production was augmented in PCAs of both males and females of old vs. young mice (Figure 2), this increase with aging was greater in males. ROS production in brain tissue was also greater in old vs. young mice. While all regions studied in the cortex illustrated this effect of advanced age for both sexes, the periventricular (noncortical) region did not. Although the reasons for this heterogenous behavior are unclear, recent findings show variability in ROS production and redox balance among different regions of the brain that correspond to their respective functions (Vinokurov et al., 2021). Our findings are consistent with systemic oxidative stress (Liguori et al., 2018) and increase vascular ROS production (Izzo et al., 2021) previously observed during advanced age. Taken together, greater ROS production from the arterial supply and surrounding parenchyma during aging may act synergistically to promote protective adaptation of the cerebral vasculature.

Oxidative stress activates multiple cellular signaling pathways including transcription factors, protein kinases, and ion channels (Kiselyov & Muallem, 2016; Kurutas, 2015). Because there were no differences between sexes in young mice for basal ROS production in brain tissue or PCAs, females exhibit a mechanism of protection that does not rely on adapting to chronic oxidative stress. Estrogen has antioxidant effects in the microcirculation (Dantas et al., 2002), which may limit the increase in secondary ROS production during H_2_O_2_ exposure (Norton et al., 2022). Complementary mechanisms of resilience in females vs. males include greater expression of the antiapoptotic protein Bcl‐2, diminished caspase 3 activity, and X‐linked inhibitor of apoptosis (Hill et al., 2011; Tsukahara et al., 2006; Vina et al., 2005). Elucidating whether such adaptations prevail in the cerebral vasculature will require further studies.

### 3.3 Reducing Ca^2+^ entry through TRPV4 channels limits cell death

Influx of Ca^2+^ from the extracellular fluid into the cytoplasm is a key determinant of cell death in PCAs (Norton et al., 2022). This rise in [Ca^2+^]_i_ leads to increases in mitochondrial Ca^2+^, ΔΨ_m_ depolarization, and cytochrome C release to initiate apoptosis (Shaw et al., 2021). The present data show that the extent of SMC death was consistently associated with an elevation of [Ca^2+^]_i_ during H_2_O_2_ exposure (Figure 3). In PCAs from young males, which were most adversely affected by H_2_O_2_, SMC death and [Ca^2+^]_i_ responses were significantly attenuated by TRPV4 inhibition. In vessels from old or female mice, cell death and [Ca^2+^]_i_ responses were minimally affected by the inhibition of TRPV4 channels. Finding that H_2_O_2_ evokes a pronounced [Ca^2+^]_i_ response in ECs (Figure S5) but not intact PCAs from females (Figure 3) indicates that Fura 2 preferentially serves as an index of SMC Ca^2+^, however, some contribution of EC Ca^2+^ to these responses cannot be ruled out. Acute H_2_O_2_ exposure increased [Ca^2+^]_i_ in endothelial tubes, with a tendency for greater Ca^2+^ influx in young vs. old. Despite this increase in endothelial [Ca^2+^]_i_, EC death and ΔΨ_m_ depolarization were minimal (Figure 1) indicating that while increases in [Ca^2+^]_i_ are necessary for H_2_O_2_‐induced apoptosis, they are not solely sufficient to induce EC death.

Finding that a TRPV4 channel antagonist reduced cell death (Figure 3), we tested whether directly activating these channels (and the ensuing rise in [Ca^2+^]_i_) would induce cell death. However, TRPV4 channel activation alone did not kill either SMCs or ECs and elicited minimal change in ΔΨ_m_, providing further evidence that oxidative stress activates pathways in addition to Ca^2+^ signaling that are necessary for cell death. The combination of H_2_O_2_ with GSK proved catastrophic for ECs (Figure 4) as reflected by the robust increase in EC [Ca^2+^]_i_ (Figure S5). Because the EC death to H_2_O_2_ + GSK was nearly complete, we investigated the underlying mechanism. Whereas EC death was greatly diminished in the absence of extracellular Ca^2+^, caspase inhibition reduced cell death by half (Figure S4). Therefore, in addition to apoptosis, additional mechanisms of EC death are activated by the combination of H_2_O_2_ and GSK that rely on increased Ca^2+^ influx from the extracellular fluid. In contrast, SMC death in response to H_2_O_2_ + GSK was less that of H_2_O_2_ alone. The reasons for this low SMC death may relate to alternative trafficking of TRPV4 channels when activated by GSK (Ambudkar, 2007). For example, prolonged exposure to the TRPV4 agonist may lead to internalization of TRPV4 channels in SMCs. However, this possibility remains to be investigated.

To further evaluate how TRPV4 function was altered by aging, we investigated the maximal [Ca^2+^]_i_ response to TRPV4 stimulation. A key finding was that the TRPV4 agonist GSK 1016790A evoked a significantly greater Ca^2+^ influx in SMCs of young vs. old PCAs and in EC tubes from both sexes (Figure 4). This response was greater in ECs vs. SMCs which may reflect why the TRPV4 agonist had such a pronounced effect on EC death when combined with H_2_O_2_. One explanation for this difference between age groups is that aging may reduce the expression of TRPV4 channels, thereby limiting Ca^2+^ influx. It is also possible that chronic oxidative stress attenuates channel activity. Despite A‐kinase accessory protein (AKAP) 150 being an activator of TRPV4 (Mercado et al., 2014; Sonkusare et al., 2014), oxidative stress can decrease TRPV4 activation by cysteine oxidation of AKAP 150 (Ottolini et al., 2021). Therefore, TRPV4 activation by oxidative stress likely occurs by a pathway other than AKAP 150, which in turn may be affected by aging.

The present results may be complicated by interactions amongst TRP channel isoforms. Although not all TRP channel subtypes can form heteromers, formation of heteromeric channels can alter their trafficking and electrophysiological properties (Clapham et al., 2001; Pires & Earley, 2017). While a variety of TRP channel subtypes can be activated by oxidative stress and/or contribute to cell death (Andersson et al., 2008; Ingueneau et al., 2009; Pires & Earley, 2017), TRPV4 and TRPC3 channels appear to be the primary isoforms responsible for apoptosis in response to H_2_O_2_ in PCAs (Norton et al., 2022). While the present study illustrates that reducing TRPV4‐dependent Ca^2+^ influx limits SMC death, ECs protect SMCs in the vessel wall from apoptosis by limiting Ca^2+^ entry through TRPC3 (Norton et al., 2022).

### 3.4 Src kinases facilitate greater cell death in PCAs of young males

Whereas H_2_O_2_ activates some TRP channels through direct oxidation of cysteine residues (Takahashi et al., 2008; Yoshida et al., 2006), TRPV4 can be activated via phosphorylation of tyrosine residues by Src kinases in response to oxidative stress (Wegierski et al., 2009). Inhibition of Src kinases reduced SMC death and attenuated the [Ca^2+^]_i_ response to H_2_O_2_ in young males (Figure 5) similar to TRPV4 inhibition (Figure 3). Further reduction of the [Ca^2+^]_i_ response to H_2_O_2_ in PCAs from old male mice treated with SU6656 did not reduce the already low level of SMC death in this group. Although the present data do not provide a direct link between Src kinase and TRPV4 activation, finding that Src kinase inhibition attenuated the rise in [Ca^2+^]_i_ during exposure to H_2_O_2_, and that HC‐067047 attenuated the [Ca^2+^]_i_ response to spermidine (Figure S7), supports the interpretation that Src kinase signaling occurs upstream from TRPV4 activation. While Src kinases contribute to oxidative stress‐induced apoptosis in epithelial cells (Chan et al., 2009), the present data provide the first evidence for a role of Src kinases affecting SMC death in response to acute oxidative stress.

Directly stimulating Src kinases with spermidine resulted in minimal cell death regardless of age or sex (Figure 6) and had minimal effects on ΔΨ_m_ in young males (Figure S6). However, spermidine evoked an increase in [Ca^2+^]_i_ which was more robust in PCAs of males vs. females. Thus, it appears that different mechanisms limit Ca^2+^ influx between sex and age during H_2_O_2_ exposure. For example, whereas female sex limits Src activity (Figure 6), advanced age limits TRPV4 activity (Figure 4) to mitigate Ca^2+^ influx and cell death in response to acute oxidative stress.

While activation of Src kinases and TRPV4 elevated [Ca^2+^]_i_, neither stimulus was capable of inducing cell death. Although spermidine can inhibit caspase 3 in neurons to limit cell death mediated by staurosporine (Yang et al., 2017), finding that activation of TRPV4 is also insufficient to induce cell death strongly suggests that H_2_O_2_ has additional effects on mitochondria which are required to link Ca^2+^ overload to apoptosis. This interpretation is supported by our results that neither GSK nor spermidine elicited depolarization of ΔΨ_m_ (Figures S4 and S6). Because increases in [Ca^2+^]_m_ lead to mitochondrial swelling and dissipation of ΔΨ_m_ (Bertero & Maack, 2018), mitochondrial Ca^2+^ channels may represent targets for additional effects of H_2_O_2_. The activation of mitochondrial Ca^2+^ channels by oxidative stress represents a promising avenue for future investigation into the coupling of Ca^2+^ overload to apoptosis.

### 3.5 Cell death impairs vasomotor control and vascular density

Acute oxidative stress plays a central role in damage from stroke and TBI (Abdul‐Muneer et al., 2015; Rodrigo et al., 2013). A hallmark of stroke is the inability of the vasculature to regulate cerebral blood flow (Markus, 2004), and therapies aimed at restoring blood flow have shown promise in experimental (Taskiran‐Sag et al., 2018) and clinical settings (Liu et al., 2014). Nevertheless, the restoration of blood flow following injury is often incomplete (Catanese et al., 2017), suggesting that damage to vascular cells may limit recovery from injury. The functional consequences of greatest cell death in PCAs from young males are demonstrated by their impaired vasoreactivity to NE and ACh following acute exposure to H_2_O_2_ (Figure 6). Our finding that preserving vasoreactivity to NE and ACh in young male PCAs with TRPV4 and Src kinase inhibition highlights these pathways as targets to maintain vasomotor control.

Consistent with the present findings, Src kinases contribute to ischemic preconditioning by limiting cell damage following ischemia (Kumar et al., 2015). Sustained oxidative stress leads to vessel rarefaction in aging (Fan et al., 2019) and the reduction in vascular density compromises cerebral blood flow and cognitive function (De Silva & Faraci, 2020). While the mechanisms of protection accompanying advanced age defined here may help limit age‐related rarefaction, prolonged exposure to ROS remains lethal to vascular cells.

### 3.6 Conclusion

The present findings demonstrate that elevated oxidative stress during advanced age is accompanied by the protection of SMCs in cerebral arteries from H_2_O_2_‐induced apoptosis by reducing Ca^2+^ influx through TRPV4 channels and attenuating the depolarization of ΔΨ_m_. Furthermore, consistent with lower Ca^2+^ influx and disruption of ΔΨ_m_ compared to age‐matched males, vessels from female mice are inherently protected from H_2_O_2_. An unexpected finding is the lethality of combining TRPV4 activation with H_2_O_2_ on ECs irrespective of age or sex. While therapeutic measures have historically focused on identifying and treating risk factors, enhancing inherent mechanisms of cellular resilience represents an alternative avenue of treatment to limit vascular damage (Gao & Galis, 2021; Taylor et al., 2022). Despite elevated ROS production in advanced age (Izzo et al., 2021), our findings indicate that cerebral circulation adapts to limit damage and preserve cerebral vascular integrity in old mice, particularly males. Targeting TRPV4 channels and Src kinases represent therapeutic applications to limit vascular damage associated with acute oxidative stress.

## 4 METHODS

### 4.1 Animal care and use

Experimental procedures were reviewed and approved by the University of Missouri Animal Care and Use Committee (Protocol #17720) and comply with ARRIVE guidelines. Male and female young (*n* = 104, 4–6 months of age, 27–32 g; Jackson Laboratories [Bar Harbor, ME, USA] RRID: IMSR_JAX:000664) and old (*n* = 88, 20–26 months of age, 32–38 g) C57BL/6J mice were studied. All mice were housed on a 12:12 h light‐dark cycle at ∼23°C with fresh water and food available ad libitum. Mice were anesthetized with ketamine and xylazine (100 and 10 mg/kg, respectively; intraperitoneal injection) to harvest brain tissue and killed by decapitation.

### 4.2 Preparation of isolated PCAs and endothelial tubes

Isolated brains were immersed in chilled (4°C) physiological salt solution [PSS, pH 7.4; containing (in mM): 140 NaCl (Thermo Fisher Scientific; Waltham, MA, USA), 5 KCl (Fisher), 1 MgCl_2_ (Sigma‐Aldrich; St. Louis, MO, USA), 10 HEPES (Sigma), 2 CaCl_2_ (Fisher), and 10 glucose (Fisher Scientific)] and pinned onto silicone elastomer (Sylgard 184®; Dow Corning, Midland, MI USA). An unbranched segment (length, ∼2 mm) of the PCA was dissected from surrounding tissue, cannulated onto heat‐polished micropipettes (outer diameter, ∼80 μm), and secured with silk suture. Cannulated arteries were placed in a tissue chamber (RC‐27 N; Warner Instrument; Hamden, CT, USA) and superfused at 3 mL/min with control PSS. Vessels were pressurized to 90 cm H_2_O (∼70 mm Hg) and maintained at 37°C throughout experiments (Norton et al., 2022).

For isolation of endothelial tubes, PCA segments (length, ∼1 mm) were placed into a round bottom test tube containing 0.62 mg/mL papain (#P4762, Sigma), 1 mg/mL dithioerythritol (#D8255, Sigma), and 1.5 mg/mL collagenase (#C8051, Sigma) in PSS and incubated for 30 min at 33°C (Norton et al., 2020; Socha et al., 2011). Vessel segments were then transferred to a 1.5 mL tissue chamber containing PSS positioned on the stage of a Zeiss GFL bench microscope for trituration to remove SMCs. Trituration pipettes were pulled from borosilicate glass capillary tubes [product no. 1B100‐4, World Precision Instruments (WPI), Sarasota, FL], heat‐polished to a tip internal diameter (ID) of ~80 μm and connected to a Nanoliter injector (WPI) for aspiration and ejection of the vessel segment. Following the dissociation of SMCs, the endothelial tube was secured at each end against the bottom of the chamber (24 × 54 mm coverslip) with blunt fire‐polished micropipettes held in micromanipulators (Figure S1).

### 4.3 Cell death

Cell death was quantified as described (Norton et al., 2019; Norton et al., 2022; Norton et al., 2023). In brief, cannulation pipettes were preloaded with PSS containing the nuclear dyes Hoechst 33342 (1 μM; Cat. #H1399, Fisher) and propidium iodide (2 μM; Cat. #P4179, Sigma). Vessels were equilibrated for 20 min in PSS containing either a pharmacological agent or its vehicle, then exposed to 200 μM H_2_O_2_ (Cat. #H1009, Sigma) in the superfusion solution for 50 min. Under these conditions, the monolayer of SMCs does not impair access of H_2_O_2_ to ECs (Norton et al., 2019). Following exposure to H_2_O_2_, the vessel was superfused with fresh PSS (without H_2_O_2_), and the lumen was perfused with PSS containing the nuclear dyes (0.1 mL/min for 10 min). Fluorescent images of Hoechst 33342 (blue) and propidium iodide (red) were acquired with a water immersion objective [40×; numerical aperture (NA) = 0.80] using appropriate filters coupled to a DS‐Qi2 camera with Elements software (version 4.51; RRID: SCR_002776) on an E800 microscope (all from Nikon). Z‐stack images were obtained from the top half of a pressurized vessel segment and live and dead cells were counted manually using ImageJ software (NIH; Bethesda, MD; RRID: SCR_003070) within a defined region of interest (ROI; 80 × 300 μm; Figure 1). EC nuclei were identified by their oval shape and orientation parallel to the vessel axis, whereas SMC nuclei were identified by their thin shape and orientation perpendicular to the vessel axis (Figure 1a).

### 4.4 Mitochondrial membrane potential

Pressurized PCAs were treated with the mitochondrial‐targeted ΔΨ_m_ fluorescent indicator tetramethylrhodamine methyl ester (TMRM, Cat. #T668, Fisher). This membrane‐permeant indicator accumulates in the matrix of mitochondria due to the electronegative potential; thus, fluorescence intensity decreases with depolarization of ΔΨ_m_ (Loor et al., 2011). Vessels were incubated with 100 nM TMRM in PSS added to the tissue chamber for 30 min without superfusion, then superfused for 10 min with PSS containing 10 nM TMRM to establish a stable baseline before adding 200 μM H_2_O_2_ to the PSS; 10 nM TMRM remained in the superfusion solution thereafter (Norton et al., 2023). Fluorescent images were acquired on an Olympus MVX10 microscope (Tokyo, Japan; RRID: SCR_018612) with an MV PLAPO 2X objective (NA = 0.5, Olympus) coupled to a megapixel CCD camera (XR/Mega10, Stanford Photonics, Palo Alto, CA, USA) at a final magnification of ∼120×. Preparations were excited at 543/22 nm and emissions were recorded at 592/40 nm. Images of TMRM fluorescence were acquired for 35 ms at 1 min intervals for 30 min and fluorescence intensity was quantified with ImageJ (NIH) in a 50 × 200 μm ROI located in the middle of a vessel following subtraction of background fluorescence. The protonophore carbonyl cyanide 4‐(trifluoromethoxy)phenylhydrazone (FCCP, 10 μM; Cat. #C6827, Sigma) added to the superfusion PSS served as a positive control to detect changes in ΔΨ_m_ (Sakamuru et al., 2012).

### 4.5 Cerebral tissue ROS production

The brain was removed from the cranium, and sections (thickness, ~1 mm) were obtained from the coronal plane to approximate the perfusion field of PCAs. The tissue section was placed in optimal cutting temperature compound (OCT; Fisher, Cat. #23‐730‐571) and frozen in isopentane placed in liquid nitrogen, then stored at −80°C. For an experiment, coronal sections (thickness, 10 μm) were obtained using a cryostat maintained at −18°C (HM550; Fisher), placed on Superfrost slides (Cat. #1255015, Fisher), and allowed to dry for ~30 min.

Slides containing coronal sections were rinsed with phosphate‐buffered saline (PBS) solution for 5 min to remove excess OCT, then blotted at the edges with a Kimwipe to remove excess PBS. The sections were incubated with 300 μL PBS containing dihydroethidium (DHE 10 μg/mL; Cat. #D23107, Fisher) (Wilhelm et al., 2016) for 10 min in the dark to prevent photoactivation of DHE, then rinsed in PBS for 5 min to remove excess DHE.

DHE is converted into the fluorescent products ethidium and 2‐hydroxyethidium in a superoxide (O_2_^**·**−^)‐dependent manner (Dikalov et al., 2008). DHE fluorescence was monitored on a Nikon E800 microscope using NIS‐Elements software in four regions of the coronal section: lower left cortex, lower right cortex, upper cortex, and a central periventricular region identified from the front of coronal sections. Images were acquired with a 20× objective using a G‐1B filter cube (excitation 546/10 nm; dichroic 565 nm; emission >590 nm) at the same exposure and gain settings for all samples. Mean fluorescence intensity was quantified in a 450 × 450 μm ROI in each section by an individual who was blinded to age or sex identity.

### 4.6 Cerebral vascular ROS production

To evaluate intrinsic H_2_O_2_ production within the vessel wall, intact pressurized PCAs were loaded with the cytosolic ROS indicator 5‐(and‐6‐)‐chloromethyl‐2,7‐dichlorodihydrofluorescein diacetate acetyl ester (DCFH; Cat. #C6827, Fisher Scientific) (Norton et al., 2020). DCFH was dissolved in DMSO and diluted to 15 μM in PSS (final DMSO = 0.5%), and a PCA was incubated in this stationary solution for 30 min. Restoring superfusion with PSS removed excess DCFH for 10 min, then fluorescence images were acquired for 35 ms at 5 min intervals over 30 min during excitation at 472/30 nm and emission recorded at 525/35 nm. Fluorescence intensity was quantified with ImageJ from a 50 × 200 μm ROI in the middle of a vessel. This optical sensor for ROS has been validated with both positive and negative controls (Norton et al., 2020).

### 4.7 Ca^2+^ photometry

To evaluate [Ca^2+^]_i_ responses in the vascular wall, which primarily reflect SMCs (Norton et al., 2022), pressurized PCAs were placed on the stage of an inverted microscope (Eclipse TS100; Nikon) and incubated in Fura 2‐AM dye (Cat. #F14158, Fisher; dissolved in DMSO and diluted to 1 μM in PSS; final [DMSO] = 0.5%) for 40 min in a stationary bath. To evaluate [Ca^2+^]_i_ responses in the endothelium (Norton et al., 2020), endothelial tubes were incubated for 30 min with Fura 2. For either PCAs or endothelial tubes, superfusion with PSS was resumed for 20 min following incubation to wash out excess dye. Fura 2 was alternately excited at 340 and 380 nm while recording emissions at 510 nm through a 20× Fluor 20 Nikon objective (NA = 0.45) using IonWizard 6.3 software (IonOptix; Milford, MA, USA; RRID: SCR_021764). Baseline fluorescence was recorded, then 200 μM H_2_O_2_ was added to the superfusion solution. [Ca^2+^]_i_ signals were recorded at 10 Hz for 30 s at 5 min intervals (to minimize photobleaching) during 50 min exposure to H_2_O_2_ and 30 min wash with PSS. For Ca^2+^ responses to HC‐076047 and spermidine, values were recorded continuously until reaching a stable plateau, maintained for 5 min, and then washed out.

### 4.8 Vasoreactivity

Vasomotor responses were studied prior to and following H_2_O_2_ exposure to determine how acute oxidative stress alters sensitivity to adrenergic vasoconstriction and endothelium‐dependent dilation. Maximal ID was recorded in Ca^2+^‐free PSS prior to each experiment using edge detection in IonWizard 6.3 software. Superfusion with control PSS was restored. Vasoconstriction to norepinephrine (NE; 10^−9^–10^−5^) and vasodilation to acetylcholine (ACh; 10^−9^–10^−5^) were evaluated in PCAs preconstricted with the EC_50_ for NE (140 nM) before exposure to H_2_O_2_ and 30 min following H_2_O_2_ washout.

### 4.9 Experimental interventions

Unless otherwise stated, all procedures were performed in vessels from young and old male and female mice. Experiments were performed in the presence of HC‐067047 (HC, 1 μM in 0.1% EtOH; Cat #4100, Tocris) to inhibit TRPV4 channels (Everaerts et al., 2010) and SU6656 (SU, 10 μM in 0.1% EtOH; Cat #6475, Tocris) to inhibit Src family kinases (Blake et al., 2000; Zhang et al., 2010), carbobenzoxy‐valyl‐alanyl‐aspartyl‐[O‐methyl]‐fluoromethylketone (Z‐VAD, 20 μM in 0.1% EtOH; Cat. #ALX‐260‐020‐M001, Enzo Life Sciences) to inhibit caspases (Fazal et al., 2005) and removal of extracellular Ca^2+^. Alternatively, intact PCAs or endothelial tubes were stimulated with the potent, selective TRPV4 agonist GSK 1016790A (GSK, 50 nM in 0.1% EtOH; Cat #6433, Tocris) to maximally activate TRPV4 channels (Ottolini et al., 2021) alone or in combination with H_2_O_2_ (200 μM). The polyamine spermidine (100 μM; Cat. #AC132740050, Fisher) was utilized to activate Src kinases (Rossini et al., 2023). Controls include respective vehicles added to PSS without the pharmacological agent.

### 4.10 Data analysis and statistics

Death of SMCs and ECs within an ROI was calculated as follows:

$$\%\text{cell death}=\left[\frac{\left(\text{Number of}\;\mathrm{red}\;\text{nuclei}\right)}{\left(\text{Number of blue nuclei}\right)}\right]\times 100\%$$

ΔΨ_m_ was assessed as the ratio of TMRM fluorescence at a given timepoint compared to baseline fluorescence (*F*/*F*_0_) and the peak change in *F*/*F*_0_ (Δ*F*/*F*_0_) following background subtraction. Values for DCFH fluorescence are expressed in arbitrary units (AU) for the change (Δ) from baseline within an ROI following subtraction of background fluorescence; linear regression was performed to determine the rate of fluorescence accumulation. Thus, ROS production was evaluated as

$$\text{Change}\;\left(\Delta \right)=\left(\text{Fluorescence}\;\mathrm{at}\;x\;\min–\text{Fluorescence}\;\mathrm{at}\;0\;\min\right)$$

where *x* represents 5‐min intervals during H_2_O_2_ exposure. Fluorescence rate is calculated as dF/d*t*, where F is fluorescence and *t* is time (min) during H_2_O_2_ exposure. After subtraction of background fluorescence recorded before dye loading, [Ca^2+^]_i_ values are expressed as the change in *F*_340_/*F*_380_ (Δ340/380) from baseline (0 min) at each 5‐min interval. Vasomotor responses were calculated as follows:

$$\%\text{spontaneous tone}=\left[\frac{\left({\mathrm{ID}}_{\max}‐{\mathrm{ID}}_{\text{base}}\right)}{{\mathrm{ID}}_{\max}}\right]\times 100$$

$$\%\text{constriction}=\left[\frac{\left({\mathrm{ID}}_{\text{base}}‐{\mathrm{ID}}_{\mathrm{NE}}\right)}{{\mathrm{ID}}_{\max}}\right]\times 100$$

$$\%\text{dilation}=\left[\frac{\left({\mathrm{ID}}_{\mathrm{ACh}}‐{\mathrm{ID}}_{\mathrm{NE},\mathrm{EC}50}\right)}{\left({\mathrm{ID}}_{\max}‐{\mathrm{ID}}_{\mathrm{NE},\mathrm{EC}50}\right)}\right]\times 100$$

where ID_max_ is maximal ID in Ca^2+^‐free PSS, ID_base_ is baseline ID in control PSS, ID_NE_ is the ID at a given [NE], ID_NE,EC50_ is the ID at EC_50_ [NE], ID_ACh_ is the response diameter at a given [ACh].

Summary data are presented as means ± SD, where *n* refers to the number of vessels (each from a different mouse) in an experimental group. Error bars are unidirectional for responses evaluated over time to improve data clarity. Power analyses using parameters *α* = 0.05, power (1−*β*) = 80% indicated a minimum group size of *n =* 4 for cell death, Ca^2+^, and vasoreactivity experiments, *n* = 5 for ROS and ΔΨ_m_. Data were analyzed using anova (Prism 10, GraphPad Software, La Jolla, CA, USA) as appropriate. When significant main effects were detected with anova, post hoc comparisons were performed using Bonferroni's tests. *p <* 0.05 was considered statistically significant.

## AUTHOR CONTRIBUTIONS

Conceptualization and study design: Charles E. Norton, Timothy L. Domeier, and Steven S. Segal. Data collection, analysis, and interpretation: Charles E. Norton, Rebecca L. Shaw, Safa, Beyoncé Dockery, Timothy L. Domeier, and Steven S. Segal. Writing—review and editing: Charles E. Norton (original draft), Rebecca L. Shaw, Safa, Beyoncé Dockery, Timothy L. Domeier, and Steven S. Segal. All authors have read and approved the final version of the manuscript and agreed to be accountable for all aspects of the work ensuring that any questions relating to accuracy and/or integrity of the work are appropriately investigated and resolved.

## FUNDING INFORMATION

This research was supported by an American Heart Association (AHA) Transformational Project Award (19TPA34850102), AHA Career Development Award (CDA931652), Alzheimer's Association Research Grant (AAGR‐NTF‐1148948), and the National Institutes of Health (R01HL136292). The funders had no role in study design, data collection or analysis, decision to publish, or preparation of the manuscript.

## CONFLICT OF INTEREST STATEMENT

The authors have no conflicts of interest.

## Supporting information


Figures S1–S8.

## Data Availability

Data supporting the findings of the present study are available at 10.7910/DVN/VCQK9F.
