# Supplementary material for: Advanced age and female sex protect cerebral arteries from mitochondrial depolarization and apoptosis during acute oxidative stress
Source: Aging Cell. 2024 Feb 21;23(5):e14110. doi: 10.1111/acel.14110 (PMC11113258; doi:10.1111/acel.14110)
Supplement: Supplementary file 1 — Figures S1–S8. [file ACEL-23-e14110-s001.pdf]

Supplemental Figures

Advanced age and female sex protect cerebral arteries from mitochondrial depolarization and apoptosis during acute oxidative stress

Charles E. Norton, Rebecca L. Shaw, Safa, Beyoncé Dockery, Timothy L Domeier, and Steven S. Segal

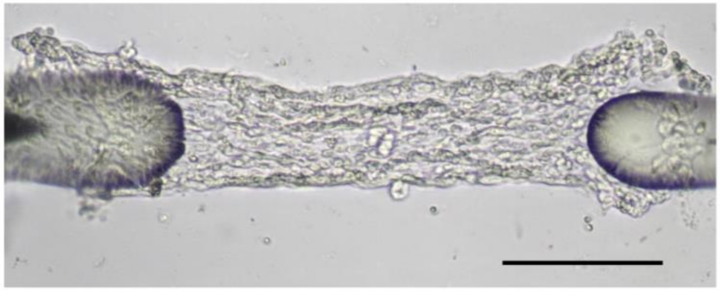

Figure S1. Brightfield image of endothelial tube. Image illustrates endothelial tube preparation secured onto a coverslip with blunted tips of glass pipettes. Scale bar = 100 μm.

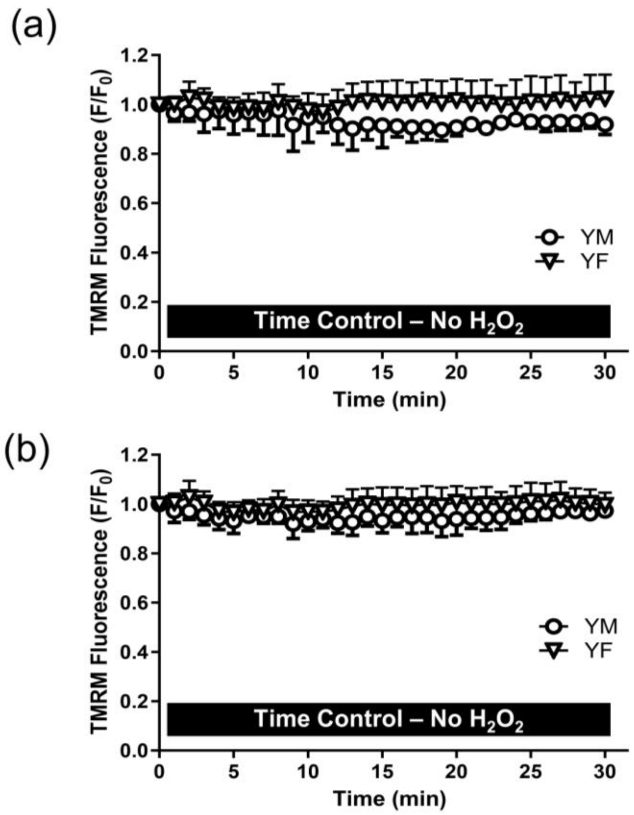

Figure S2. TMRM fluorescence remains stable over time. Time controls in the absence of H_2O_2 illustrate that $\Delta\Psi_m$ remains stable for the duration of an experimental protocol in (a) pressurized PCAs and (b) endothelial cell tubes from young males (YM) and young females (YF). Data are means ± SD; *n*=5-7 vessels/group. No significant differences were detected. Statistics: one-way ANOVA with Bonferroni post hoc tests.

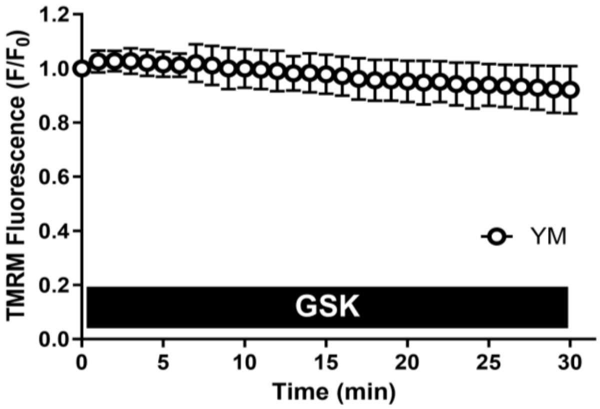

Figure S3. TRPV4 activation evokes minimal ΔΨ$_m$ depolarization. Changes in ΔΨ$_m$ during GSK (50 nM) exposure in PCAs from young males (YM). Data are means ± SD; *n*=5 vessels.

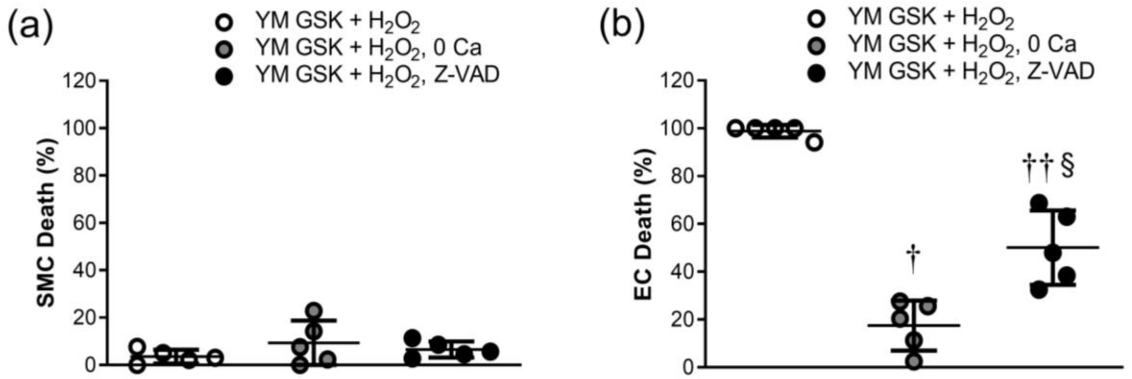

Figure S4. Role for Ca^{2+} influx and caspases in cell death from TRPV4 activation during oxidative stress. (a) SMC death and (b) ED death in PCAs from young males (YM) exposed to GSK+H_2O_2, GSK+H_2O_2 in the absence of extracellular Ca^{2+} (0 Ca^{2+}), or GSK+H_2O_2+Z-VAD (caspase inhibitor, 20 µM) for 50 min. Data are from individual experiments with means ± SD; *n*=5 vessels/group. For ECs: $^{\dagger}P<0.05$, GSK + H_2O_2, 0 Ca *vs*. GSK + H_2O_2. $^{\dagger\dagger}P<0.05$, GSK + H_2O_2, Z-VAD *vs*. GSK+H_2O_2. $^{\S}P<0.05$, GSK+H_2O_2, Z-VAD *vs*. GSK+H_2O_2, 0 Ca^{2+}. Statistics one-way ANOVA with Bonferroni post hoc tests.

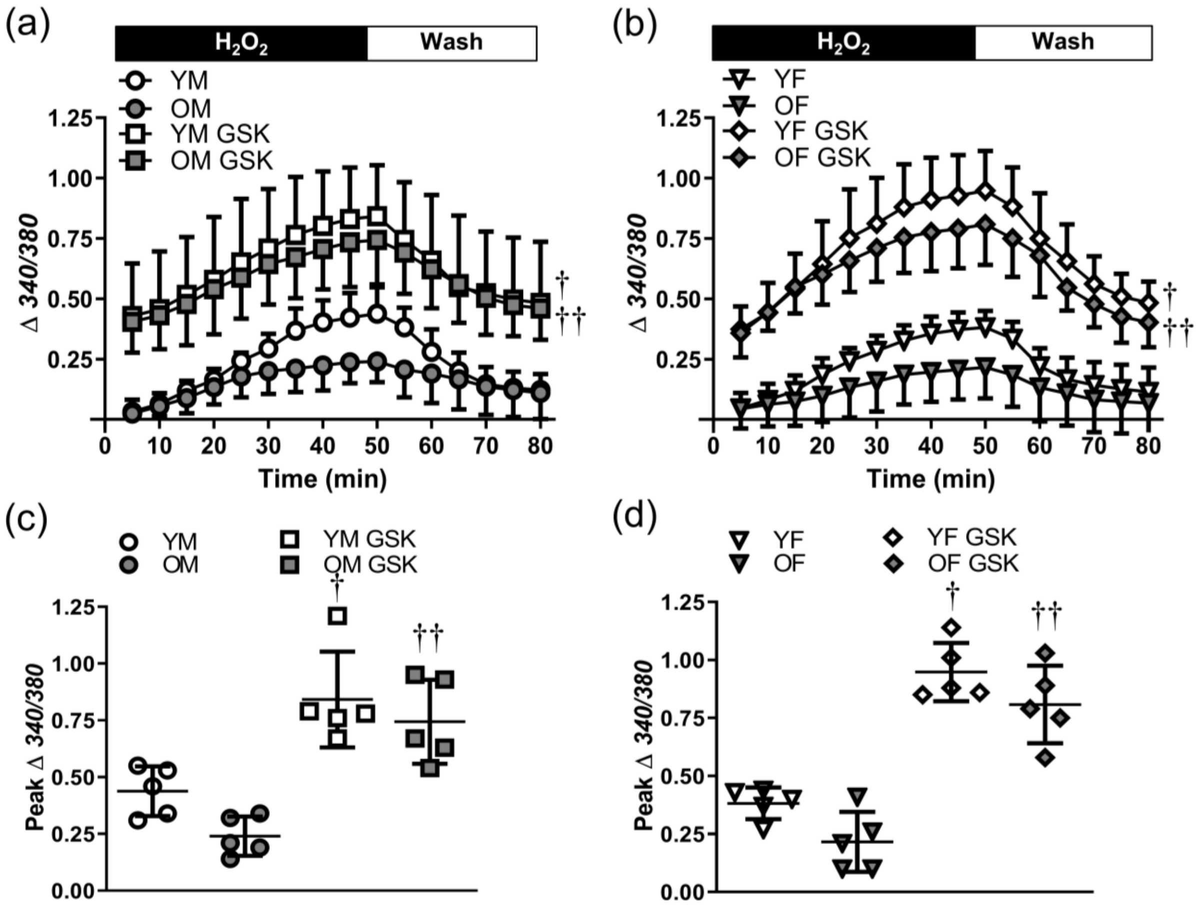

Figure S5. TRPV4 activation augments increases in EC Ca^{2+} during acute oxidative stress. (a) $[Ca^{2+}]_i$ responses (Fura 2 fluorescence) of endothelial tubes from young males (YM) and old males (OM) during 50 min H_2O_2 or H_2O_2+GSK followed by 30 min wash in control PSS. (b) $Ca^{2+}]_i$ responses for young females (YF) and old females (OF). Peak changes in $[Ca^{2+}]_i$ in (c) YM and OM and (d) YF and OF. Data are from individual experiments with means ± SD; *n*=5 per group. †P<0.05, GSK+H_2O_2 *vs*. H_2O_2 in young. ††P<0.05, GSK+H_2O_2 *vs*. H_2O_2 in old. Statistics: two-way ANOVA with Bonferroni post hoc tests.

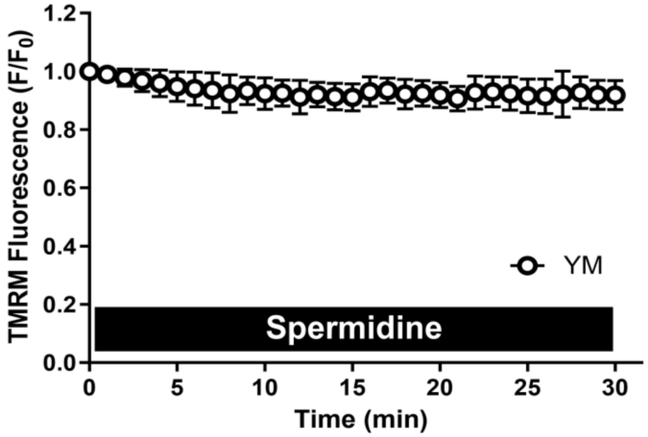

Figure S6. Activation of Src kinases elicits minimal changes in $\Delta\Psi_m$. Changes in $\Delta\Psi_m$ during spermidine (100 µM) exposure in PCAs from young males. Data are means ± SD; *n*=5 vessels.

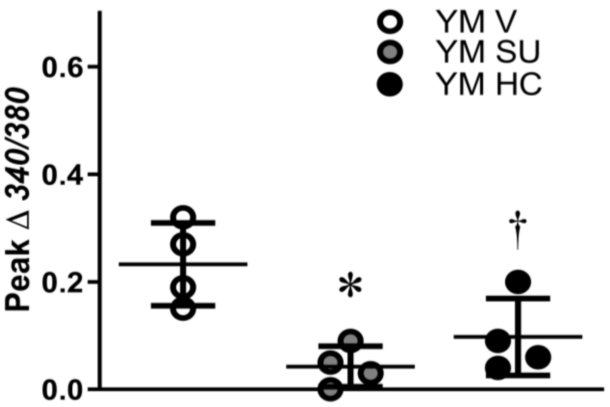

Figure S7. TRPV4 Inhibition attenuates Src-dependent Ca^{2+} entry. Peak changes in $[Ca^{2+}]_i$ to spermidine (100 µM) in the absence (vehicle; V) and presence of the Src kinase inhibitor SU6656 (SU, 10 µM) and the TRPV4 channel inhibitor HC-067047 (HC; 1 µM) in PCAs from young males (YM). Data are from individual experiments with means ± SD; *n*=4 vessels/group. *P<0.05, SU *vs*. V. †P<0.05 HC *vs*. V. Statistics: one-way ANOVA with Bonferroni post hoc tests.

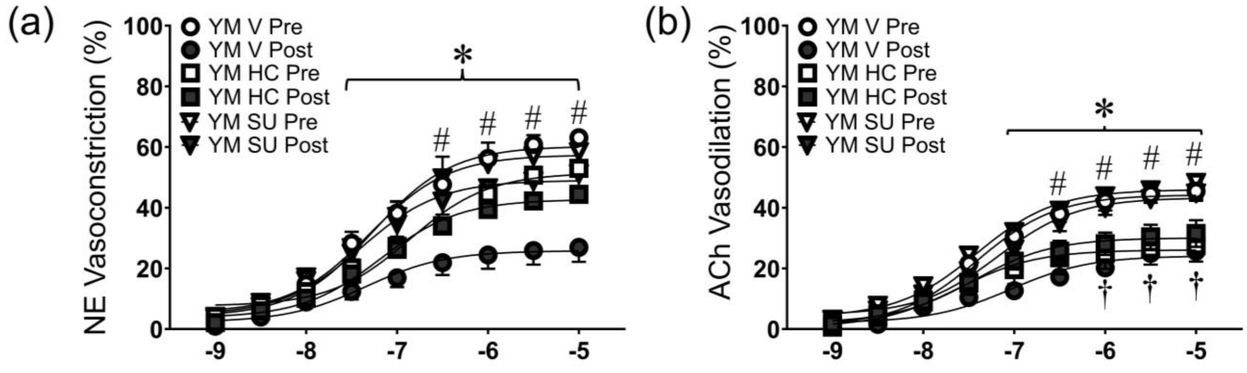

Figure S8. Src and TRPV4 inhibition preserve vasomotor function in young males. (a) Concentration-response curves for vasoconstriction to NE in PCAs from young males (YM) before (pre) and after (post) H_2O_2 exposure in the absence (vehicle; V) or presence of the TRPV4 channel inhibitor HC-067047 (HC, 1 µM) or the Src kinase inhibitor SU6656 (SU, 10 µM). (b) Concentration-response curves for vasodilation to ACh in PCAs from YM before and after H_2O_2 exposure in the absence or presence of HC-067047 or SU6656. Data for YM V Pre and YM SU Pre overlie YM SU Post and data for YM HC Pre and YM HC Post overlie YM V post. Data are means ± SD; *n*=4 vessels/group. *P<0.05, pre *vs*. post in V. $^{\#}P<0.05$, SU post *vs*. V post. $^{\dagger}P<0.05$ HC pre *vs*. V pre. Statistics: two-way ANOVA with Bonferroni post hoc tests.
